# Understanding the Psychosocial and Parenting Needs of Mothers with Irritable Bowel Syndrome with Young Children

**DOI:** 10.3390/children7080093

**Published:** 2020-08-07

**Authors:** Lexa K. Murphy, Tanera R. van Diggelen, Rona L. Levy, Tonya M. Palermo

**Affiliations:** 1 Center for Child Health, Behavior and Development, Seattle Children’s Research Institute, PO Box 5371, M/S CW8-6, Suite 400, Seattle, WA 98145, USA; Tanera.VanDiggelen@seattlechildrens.org (T.R.v.D.); tonya.palermo@seattlechildrens.org (T.M.P.); 2 Department of Social Work, University of Washington, Seattle, WA 98145, USA; rlevy@uw.edu; 3 Department of Anesthesiology & Pain Medicine, University of Washington, Seattle, WA 98145, USA

**Keywords:** irritable bowel syndrome, chronic pain, parenting, children

## Abstract

Women of childbearing age experience the highest prevalence of irritable bowel syndrome (IBS), yet little is known about their psychosocial and parenting needs, which may influence their children’s experience of future gastrointestinal or pain-related conditions. The aims of this study were to conduct qualitative interviews to understand the psychosocial and parenting needs of mothers with IBS who have young school-age children, and to assess mothers’ potential interest in and acceptability of a preventive parenting intervention program. Ten mothers with IBS who have young (age 5–10), healthy children were interviewed. Interviews were coded with thematic analysis and three themes were identified: (1) Guilt about how IBS impacts children, (2) Worry that children will develop IBS, and (3) Already on high alert for children’s health. All mothers expressed interest in an Internet-based preventive intervention and identified tools and strategies they would want included. Results demonstrate that mothers experience guilt about how IBS has impacted their children in their daily lives, concern that they need to pay attention to children’s early signs and symptoms that could indicate gastrointestinal problems, and worry about children developing IBS in the future—suggesting that a preventive intervention may address important concerns for this population.

## 1. Introduction

Women of childbearing age experience the highest prevalence of irritable bowel syndrome (IBS) with approximately 1 in 10 women reporting IBS symptoms [1,2]. IBS is a chronic gastrointestinal condition associated with chronic pain and impairments in health-related quality of life, psychological functioning, and work and social functioning [3,4,5,6,7,8,9,10]. In the United States, 2.4 to 3.5 million doctor visits each year are due to IBS [11], and the direct healthcare costs due to IBS are estimated at $1.3 billion per year [12]. Despite the high prevalence of IBS in women of childbearing age, and the well-documented impact of IBS on daily functioning, limited information is known about the psychosocial and parenting impacts experienced by mothers with IBS who have young children.

Prior studies have shown that mothers with a range of chronic pain conditions (e.g., fibromyalgia, musculoskeletal pain, headache) report worry about their own pain, concern about their ability to parent, and worry about whether their children will develop chronic pain [13,14,15]. In a study in mother-adolescent dyads, mothers with chronic pain reported engaging in protective responses when their teen complained of pain [13]. However, to our knowledge, no prior studies have characterized the parenting needs and concerns of mothers with IBS who have young school-age children. This age group is particularly relevant as pain complaints such as abdominal pain often begin during the school-age period [16], representing a critical opportunity for the development of coping skills for pain and gastrointestinal (GI) symptoms [17]. Furthermore, during this developmental period, children learn an expanded repertoire of coping skills, begin to intentionally select and deploy specific skills for specific stressors, and look to parents as primary social models for coping [18].

Understanding the needs of this group is also important as the impact of parents’ IBS extends to children. Youth whose mothers have IBS or other idiopathic abdominal pain conditions are at over 2x greater risk for developing a chronic pain condition in adolescence [19,20], have significantly more healthcare visits and higher healthcare costs [21], and are at risk for chronic pain and GI symptoms that extend into adulthood [22]. A study comparing rates of idiopathic chronic abdominal pain in monozygotic and dizygotic twins and their parents found evidence that the contribution of learning processes to the etiology of IBS in children was greater than that of genetics [23]. According to social learning theory, parents influence the development of health behaviors and beliefs in their children via direct and indirect learning [24,25]. In the context of abdominal pain conditions such as IBS, maternal modeling of maladaptive pain behaviors, pain catastrophizing, as well as solicitous or protective responses to child pain are all posited to increase risk for pain and disability in children [21,26,27,28]. However, to date, these processes have largely been examined in families in which both mothers and adolescent children have established chronic pain conditions. Identifying parenting needs of mothers with IBS with young, healthy school-age children may present important opportunities to develop preventive interventions.

Therefore, the aim of this study was to conduct qualitative interviews to understand the psychosocial and parenting needs of mothers with IBS who have young school-age children (age 5 to 10). The second, exploratory aim of this study was to assess mothers’ potential interest in and acceptability of a preventive parenting intervention program.

## 2. Methods

### 2.1. Participants and Procedure

Participants were recruited through the community via social media advertisements and flyers posted in public places (e.g., library, community centers). Those interested in the study were directed to complete a brief screening form on REDCap (Research Electronic Data Capture), a secure web-based data collection platform, and then contacted by phone to complete eligibility screening and consent. Inclusion criteria were: the *mother* (a) has been diagnosed with IBS and has had abdominal pain occurring at least weekly during the past 6 months, (b) is the parent primarily responsible for caring for the child, and (c) does not have another significant medical condition (e.g., cancer) requiring chronic medical treatment. The mother has a *child* who (a) is 5 to 10 years old, (b) has never received medical care for idiopathic abdominal pain and has never met criteria for a chronic digestive disorder (e.g., functional abdominal pain or IBS) and (c) does not have a developmental disability or a chronic disease (e.g., cancer) requiring chronic medical treatment. After initial screening and informed consent, participants completed semi-structured interviews by phone, described below, and received a $50 gift card to thank them for their time.

Twenty-three potential participants completed the initial REDCap screener. Eight were ineligible (three had children who already received medical attention for idiopathic abdominal pain, three had Crohn’s Disease, one had a child <5 years old, and one mother was never diagnosed with IBS) and five were unable to be reached after filling out the initial interest form. Ten mothers were enrolled in the study and completed all study procedures. Participants in this sample were White, ranged in age from 30 to 45, and represented a range of occupations and household constellations (married, divorced, never married). Participants had between one to two children aged 5 to 10 years old, and selected the youngest child to consider in the interview. All mothers reported IBS symptoms that occurred at least weekly; all participants reported abdominal pain and diarrhea, 40% reported constipation, 30% cramping, and 40% bloating. Years since diagnosis ranged from 2 to 24 (*M* = 13.9). Participants reported having tried the following treatments that were recommended for IBS management: dietary (*n* = 9 avoiding individual trigger foods or low-FODMAP diet), medication (*n* = 7 over the counter medications, e.g., probiotic, stool softener, antacid, antispasmodic; *n* = 2 prescription medications, i.e., anti-depressant, opioid), psychological (*n* = 1), and complementary or integrative (*n* = 4, e.g., yoga, exercise, meditation).

### 2.2. Measures

Participants completed a semi-structured interview by phone with the first author ranging in duration from 17 min to 37 min. Consistent with inductive reflexive thematic analysis [29,30], we did not aim to saturate our sample but instead stopped data collection when we had collected sufficiently rich data to address our aims (*n* = 10 mothers). Interviews were audio-recorded and transcribed orthographically. Identifying information in the transcripts was removed prior to coding.

The qualitative interview prompts were created by the study team based on principles of qualitative analysis [29] and prior literature [31,32,33,34,35,36] for the purposes of this study. Participants were asked: *How long have you had IBS and abdominal pain and what has it been like for you? How has IBS affected your life? Some mothers with IBS tell us that they worry about their children developing a pain problem like IBS in the future and others do not—can you tell us what this has been like for you?* Following these questions, participants were asked about their interest in/acceptability of a web-based preventive intervention, and were specifically asked about the acceptability of potential intervention components with a combination of open-ended and forced choice (yes/no) questions. These prompts were modeled after principles of user-centered design in intervention development [37] and adapted from a former needs assessment interviews [31,32].

### 2.3. Data Analysis

Qualitative interview transcripts were analyzed with the qualitative analysis software NVivo [38] according to principles of reflexive thematic analysis [29]. Reflexive thematic analysis is a flexible approach developed by Braun and Clark conducted in six stages: familiarization, coding, generating initial themes, reviewing themes, defining/naming themes, and writing. In the familiarization phase, two primary coders (LM & TV) reviewed the collected transcripts. During the coding phase, TV again reviewed all transcripts and began to identify potential codes, which were reviewed by LM to ensure reliability. Codes were then reviewed iteratively for generalizability. Following the coding phase, LM and TV met to review codes and discuss initial themes. All authors then discussed final definitions and themes prior to writing the results. Responses to questions about interest in/acceptability of a preventive intervention were coded using principles of directed content analysis [39]. The coding team, LM & TV, discussed application of codes and reached consensus through discussion. Responses to yes/no prompts were tallied and simple descriptives (percentages) were computed to summarize responses.

Quality of coding was addressed through the use of Braun & Clarke’s [29] 15-point Checklist of Criteria for Good Thematic Analysis and Yardley’s [40,41] Quality Principles. This includes transcription (using orthographic transcribing), coding processes (six stages reviewed above), sensitivity to context (contextualizing the research in relevant literature, sensitivity to participants’ perspectives by allowing participants to “tell the story” of their IBS), commitment and rigor (data collection, depth of analysis, professional engagement with the topic), and transparency and coherence (fit between research question and data collection and analyses, researcher as active). Regarding professional expertise of the coding team, the primary coders consisted of a postdoctoral-level researcher who has research experience in pediatric pain and preventive parenting interventions and clinical experience working with youth and parents with IBS, as well as a post-baccalaureate research coordinator who has experience supporting research in pediatric pain and parent training programs. The coding team also consisted of faculty researchers with extensive experience conducting observational and interventional research with parents and children with chronic pain disorders including IBS.

## 3. Results

Three themes pertaining to psychosocial and parenting needs were identified from qualitative interviews: (1) Guilt about how IBS impacts children, (2) Worry that children will develop IBS, and (3) Already on high alert for children’s health. Each theme consisted of two to three codes. See Table 1.

### 3.1. “I Can’t Care for Them and Care for Myself at the Same Time”: Guilt About How IBS Impacts Children

Participants expressed guilt about the impact their IBS has had in their daily lives on their children. The first code within this theme is: *IBS Gets in the Way of Being a Good Mom*. Participants describe feeling pulled away from their children when IBS flares and express guilt that IBS symptoms have kept them from being the type of mom they wanted to be. For one mother, IBS flares kept her from being able to do “active play” that her children love, and she worried that her children might interpret this as “avoiding” them on purpose. For another mother, IBS kept her from providing “attention” and “love” to her child. She explained, “like my daughter wanted to lay on top of me and cuddle, and my stomach just hurt to the touch, and I was like ‘I can’t. Like I love you, but I can’t cuddle you right now. Every part of my body hurts from being ill.’” Another felt “completely useless” with her children during flares because “I can’t walk around, I can’t sit up, I can’t engage in activities, I can barely move.” Another mother provided an apt summary for this dilemma: “I can’t care for them and care for myself at the same time.”

The second code within this theme is *Missing Out on Important Family Moments.* Several participants recalled important family events that they missed out on with both guilt and sadness.

One mother described taking a family vacation for a holiday weekend, having an IBS flare, and then staying inside while her husband and child explored the city. She explained “there is that impact of being isolated from my family because I can’t function,” Another participant described the difficulty of planning family trips knowing that IBS may interfere; she has often had to cancel plans with children at the last minute explaining, “no, not today.” Some mothers believed that their IBS also caused their children to miss out on important events themselves. One mother had to repeatedly cancel her daughter’s birthday party due to IBS flares, resulting in her daughter feeling “really upset” because “she doesn’t understand what’s going on.” Implicit across these accounts is the belief that IBS results in lost family moments.

*Children Shouldn’t Have to Carry IBS Burden* is the final code within this theme. This code taps feelings of guilt related to both what children witness when IBS is flaring as well as how much children worry about their mother’s health. One mother explained, “she’s seen me laying on the bed, you know pushed up with the pillow by my stomach” and now worries that this image is “in the back of her mind all this time.” The same mother also worried what would happen if she was out with her child and had a sudden flare: “I wouldn’t want her to be embarrassed if I had an accident.” Several participants described occasions when symptoms were so bad their children started caring for them, checking in on them while they were resting or in the bathroom. One participant said, “They just want to take care of me though, and kids shouldn’t have to take care of adults. That’s not, not good for them.” Another mother expressed guilt that her son has witnessed some of her worst flares, and has struggled to explain her symptoms to him in a way that won’t worry him further: “It’s hard for me to articulate… We’re very open and I communicate. But you know what, I’m not an expert in this. I know what I’m going through, but I try to find a way to communicate that’s not going to scare him.” Another mother explained that, even though she thinks that her children worry about her, “they just don’t talk about it.” Across these accounts, it is evident that mothers are unsure about how best to explain symptoms to their children to keep them from worrying.

### 3.2. “I Would Not Want Her to Turn into Me”: Worry that Children Will Develop IBS

Participants also expressed a future-oriented worry that their children may grow up to develop IBS themselves. Within this theme, the first code is *Worry That Children Are at Heightened Risk for IBS*. Participants expressed, with varying degrees of certainty, that because they have IBS their children also carry a genetic risk for IBS. One mother shared, “in my family there’s a pretty strong propensity towards stomach problems—IBS, acid reflux, ulcers—and that, that concerns me, I keep an eye out for it.” Another mother expressed worry for her child given the high rates of GI disorders in her family: “My mom, certainly, my brother, my grandfather and my great-aunts, I know all had issues. And so it does, it does concern me that I have girls who may go through the same thing.” One mother explained that this worry is strongest when visiting the pediatrician, and she is just “waiting” for a diagnosis to come. In addition to worry about risk, participants expressed that they would feel responsible or at “fault” if their child did develop IBS. One participant explained, “I would not want her to turn into me.”

The second code, *Worry That Developing IBS Will Impact Children’s Future*, moves beyond expressing worry that their child will develop IBS, and more specifically captures fears related to how this will limit their child’s life in the future. Mothers anticipated ways that IBS may negatively impact their children’s future nutritional health, social life, and employment. One mother said, “if they have the same symptoms I have throughout their life, it would be terrible.” Drawing on her own experience with IBS and assuming her child’s would be the same, another mother said, “it doesn’t feel good, but it also stops you from doing things, and it’s embarrassing.” Another mother expressed worry more broadly about her child’s health: “When you have a chronic condition…it is overwhelming just to have the history and knowledge of things that could go wrong.”

### 3.3. “I Watch Very Carefully”: Already on High Alert for Children’s Health

The final theme reflects an assumption that mothers with IBS need to pay close attention to children’s symptoms and work to optimize their child’s health. Distinct from other themes, this is focused on what participants feel they need to do now in the present. The first code, *Looking for Early Signs and Symptoms*, reflects a need to pay close attention to children’s pain and GI symptoms. Some participants engage in monitoring; for example, one mother said, “I watch very carefully to see, like if he has any issues, without projecting like, ‘hey how’s your stomach?’” Other mothers take a more active role and describe questioning their child when they notice a change in behavior: “[Child name], does your tummy hurt?” Similarly, another mother described frequently soliciting information from her child: “I ask her all the time, ‘Do you have diarrhea? Does your tummy hurt?’” This code also reflects an assumption that, if children may be developing IBS, there is a need to catch symptoms early and seek medical attention. For example, one mother said, “It could be something during the next time I bring him to the doctor that I bring up…could this be something that’s starting early for him as well?”

Perhaps grounded in the assumption that they need to start working to safeguard their children’s health now, the second code in this theme is *Need to Optimize Children’s Diet*. Participants describe putting children on the same IBS-friendly diet that they have developed for themselves in order to improve their child’s health. Some participants assume a more preventative stance, with an intent to optimize gut health: “I try to make sure that they are set up for a good like microbiome in their gut by having a wide range of, of fruits and veggies and healthy foods and whole grains.” Others focus on eliminating foods that they believe have precipitated their own IBS flares, or staying away from “the trigger foods”. One mother shared concerns about her daughter developing IBS with a pediatrician, and even though he told her “I don’t think that’s what’s going on,” she has already put her on a special diet because “there are maybe certain things she needs to avoid.” Across participants, there is an assumption that an improved diet can either reduce risk for or reduce severity of IBS in the future for their children.

### 3.4. Interest in Preventive Intervention

All participants expressed interest in a program that could help mothers with IBS reduce the likelihood that their children will grow up to have problems with IBS and pain. Additionally, all participants expressed interest in an Internet-delivered program. In response to an open-ended question about tools or strategies that mothers would want to be included in a potential program, responses were coded into three categories: skills, information, and social support. Forty percent expressed an interest in a social support component. Fifty percent expressed interest in skills; specifically, this included skills for parenting (10%), skills for communication with family members about IBS (10%), and skills to teach to children (20%). Sixty percent expressed interest in information; specifically, this included information related to medical resources (40%), diet/nutrition (30%), IBS risk in children (20%), and tailoring skills to diverse families/children (10%). Subsequently, participants were asked to respond yes or no as to whether they would be interested in specific intervention components: Ways of managing pain that would allow you to do more of the activities you want to do (100% yes), ways to solve problems that come up related to IBS and parenting (100% yes), ways to cope with stress in your life related to IBS (90% yes), and communication tools you can use with your children (90% yes).

## 4. Discussion

This study reveals significant psychosocial and parenting needs of mothers with IBS with young school-age children. Based on qualitative interviews, mothers experience guilt about how IBS has impacted their children in their daily lives, concern that they need to pay attention to children’s early signs and symptoms that could indicate GI problems, and worry about children developing IBS in the future. These themes demonstrate that mothers with IBS care a great deal about how their IBS impacts their children and suggest that a preventive intervention to reduce risk of pain in children may be relevant and address important concerns for this population.

Prior qualitative studies that have assessed the healthcare needs of women with IBS have identified themes related to the unpredictability of IBS symptoms and the impact of symptoms on multiple domains of daily life [33,34,35,36]. This is the first qualitative study to purposefully sample mothers with IBS to understand the impact on parenting. Consistent with prior studies, participants described burden and worry associated with the unpredictability of IBS symptoms. However, our study extends prior findings by revealing important concerns that mothers have regarding their children—both in terms of how IBS has impacted their parenting in their day-to-day activities and how it might burden children in the future should their children develop IBS.

We focused on mothers of school-age children, as this represents an important period in which children not only develop an expanded repertoire of coping skills, but also begin to intentionally select and deploy specific skills for specific stressors including painful events [18]. Critical in the development of such skills are social models, and the most salient social models during this period are parents [18]. This study therefore provides important insights into the experiences of mothers with IBS with school-age children, and the social context in which children are learning how to cope with pain, GI symptoms, and stress.

Because observing a parent in abdominal pain may influence the development of children’s pain coping skills [17,21,28], our results highlight both strengths and challenges that may be important to consider in the context of a preventive intervention. In regard to strengths, mothers in this study were aware that their children may be at heightened risk for the development of IBS, and many of them have already adopted a preventive stance by trying to optimize their child’s health as best they can. Across interviews, mothers also demonstrated a desire and motivation to better manage their own GI symptoms in order to spend more time with their children. Furthermore, several participants described intentionally spending time away from children when experiencing flares, perhaps reflecting an assumption regarding the importance of modeling wellness behavior. In addition, mothers expressed a desire to explain IBS symptoms to their children in a way that would keep them from worrying or engaging in caretaking behavior.

Results also revealed challenges that provide important social context about the impact on parenting. Some participants described interference or avoiding daily activities when in pain, which may model negative pain behaviors to their children. Several participants described negative thought patterns including ruminating about how IBS symptoms have impacted parenting, helplessness about how symptoms impact children, and worries about children’s health. Finally, behaviors described in the code *Looking for Early Signs and Symptoms* may represent potentially solicitous or protective responses to children’s pain complaints such as asking repeatedly about stomach pain, which is considered a less adaptive parenting behavior. Participants also described modifying children’s diets to try to optimize health and reduce IBS risk. While parents’ intentions were to reduce risk for GI symptoms, it is important to note that, in some cases, restrictive diets may lead to other problems such as disordered eating later in childhood and adolescence [42,43,44]. Although previous quantitative and qualitative studies have documented social risk factors in parent-child dyads in which both have a chronic pain or gastrointestinal condition [13,21], this study highlights that it is also important to understand parenting experiences in mothers with young, healthy children. Taken together, pain modeling, thought patterns, responses to child pain, dietary changes, and family communication could all represent important considerations in the context of a preventive intervention.

Broadly, the results from this study highlight that participants are concerned about their children’s heightened risk of developing IBS and believe it is important to develop their own preventive or mitigating strategies. All participants expressed interest in a potential preventive intervention that could help reduce their children’s likelihood of developing IBS and pain in the future. All participants were also accepting of an Internet-delivered intervention. When asked, mothers in this study specifically expressed a desire for tools and strategies focused on skills, information, and social support for parenting with IBS. Taken together, these results suggest that a preventive intervention may address an important need and be well-received.

In previous research, social-learning cognitive-behavioral interventions delivered in person and remotely have consistently shown significant improvements in parent outcomes (pain behaviors, pain catastrophizing, parenting behaviors) and child outcomes (pain symptoms, disability, quality of life) compared to control conditions [45,46,47,48]. However, prior trials have focused on treating pediatric pain and disability after it has already developed. To date, there have been no *preventive* psychological interventions for children at risk for the development of chronic pain and GI disorders. Given both increased risk for children of mothers with pain conditions as well as the high cost of IBS [3,4,5,6,7,8,9,10,11,12], there is a need for preventive interventions for this population. We expect that mothers may benefit from intervention strategies targeting both IBS symptom management and parenting. For example, IBS self-management skills may include cognitive strategies, problem-solving, behavioral activation, and adaptive pain coping skills that aim to reduce functional impairment. Parenting specific strategies may include communication skills (e.g., identifying and challenging catastrophizing cognitions about children) and building adaptive behaviors for coping with stress and physical symptoms (e.g., scheduling positive activities with children, and modeling adaptive pain behaviors for children).

Findings should be considered in light of the following limitations. First, participants were recruited from a single metropolitan area and were all white, which limits generalizability of findings. Given that we employed community-based recruitment, diagnoses and symptoms are based on self-report and IBS phenotypes are not specified. Each participant in this sample had symptoms of weekly pain and diarrhea, which likely impacted the qualitative themes for this study, particularly codes that stemmed from the unpredictability and urgency of their IBS symptoms. In addition, we chose to use inductive thematic analysis, which focuses on participants’ experiences but does not necessarily generate or confirm existing social-learning theory.

There are also important points to consider for future studies. First, we focus purposefully on mothers given the prevalence of IBS in women of childbearing age and the extant literature on intergenerational transmission of illness behavior between mothers and children [19,20]. However, it is also important for future studies to examine parenting and psychosocial needs of fathers with IBS, as well as households where both parents may have GI conditions. While this sample included mothers who experienced functional impairment associated with their IBS, intervention development may also be informed by the experiences of mothers who do *not* experience their IBS symptoms as impairing, pointing to specific resiliency factors that could be targeted in an intervention as well. Finally, future research would benefit from collecting information on participants’ cultural background and experiences, which would impact parenting assumptions and practices and would need to be considered in the context of a parenting intervention.

In summary, results from this qualitative study demonstrate that mothers with IBS with young children experience guilt about how their symptoms have impacted children, worry that their children will go on to develop IBS in the future, and are already on high alert for symptoms in children. This highlights important parenting and psychosocial needs that could be addressed in a future preventive intervention.

## Figures and Tables

**Table 1:** Themes and codes.

Theme	Codes
“I can’t care for them and care for myself at the same time” *Guilt about how IBS impacts children*	IBS gets in the way of being a good mom
	Missing out on important family moments
	Children shouldn’t have to carry IBS burden
“I would not want her to turn into me” *Worry that children will develop IBS*	Worry that children are at heightened genetic risk
	Worry that developing IBS will impact child’s future
“I watch very carefully” *Already on high alert for children’s health*	Looking for early signs and symptoms
	Need to optimize children’s diet
